# Impact of Aging on the Accuracy of 3D-Printed Dental Models: An In Vitro Investigation

**DOI:** 10.3390/jcm9051436

**Published:** 2020-05-12

**Authors:** Tim Joda, Lea Matthisson, Nicola U. Zitzmann

**Affiliations:** Department of Reconstructive Dentistry, University Center for Dental Medicine Basel, University of Basel, 4058 Basel, Switzerland; lea.matthisson@unibas.ch (L.M.); n.zitzmann@unibas.ch (N.U.Z.)

**Keywords:** rapid prototyping, 3D printing, accuracy, dental materials science, digital workflow

## Abstract

The aim of this in vitro study was to analyze the impact of model aging on the accuracy of 3D-printed dental models. A maxillary full-arch reference model with prepared teeth for a three-unit fixed dental prosthesis was scanned ten times with an intraoral scanner (3Shape TRIOS Pod) and ten models were 3D printed (Straumann P-Series). All models were stored under constant conditions and digitized with a desktop scanner after 1 day; 1 week; and 2, 3, and 4 weeks. For accuracy, a best-fit algorithm was used to analyze the deviations of the abutment teeth (GFaI e.V Final Surface^®^). *Wilcoxon Rank Sum Tests* were used for comparisons with the level of significance set at α = 0.05. Deviation analysis of the tested models showed homogenous intragroup distance calculations at each timepoint. The most accurate result was for 1 day of aging (3.3 ± 1.3 µm). A continuous decrease in accuracy was observed with each aging stage from day 1 to week 4. A time-dependent difference was statistically significant after 3 weeks (*p* = 0.0008) and 4 weeks (*p* < 0.0001). Based on these findings, dental models should not be used longer than 3 to 4 weeks after 3D printing for the fabrication of definitive prosthetic reconstructions.

## 1. Introduction

Digitalization is en vogue: *#WhatCanBeDigitalWillBe*. Therefore, it is not surprising that the demands of clinicians and patients are also changing in the field of dentistry. Scan technology has opened the possibility to digitize the patient’s dental situation: either lab-side scanning of conventional gypsum casts or directly chairside with an intraoral scanning device (IOS). With both methods, the patient-specific situation can be captured optically and stored as a three-dimensional (3D) surface file, namely a standard tessellation language (STL) file [1]. Scan technology is currently of great interest in all dental disciplines, in particular in prosthodontics for the manufacturing of fixed dental prostheses (FDP) [2].

IOS enables fully digital chairside workflows, incorporating computer-aided-design and computer-aided-manufacturing (CAD/CAM) without any physical models [3]. IOS meets the ubiquitous trend of digitalization in the society, supports more convenient treatments [4], and will successively displace conventional impression taking in dentistry [5]. Whenever possible, complete digital workflows will be used in dental medicine in the future [6].

The typical IOS domain has been single-unit restorations. Complete digital workflows have been proven for tooth- and implant-supported monolithic single-unit restorations, especially in posterior sites [7,8]. From an economic point of view, clinical and technical protocols for single crowns can be streamlined to achieve time-efficient therapy outcomes with a reasonable cost–benefit ratio and a high quality of CAD/CAM-processed restorations [9,10].

At present, not all prosthetic indications can be addressed with model-free workflows, i.e., manually veneered multi-unit FDPs. Even though technical progress is rapid and the combination of IOS and laser-melting seems to be promising for CAD/CAM processing of frameworks of removable partial dentures (RPD), a dental model is still required for the finalization of the RPD [11,12]. With the increase in performance of IOS devices, however, the desire to expand the range of indications from single crowns to more complex prosthetic reconstructions including removable restorations with edentulous mucosal tissues has grown [13]. Clinical and technical protocols (combining IOS and dental model fabrication) are required to cover such complex indications in a digital workflow. Rapid prototyping is a technique to construct and build any geometry using 3D printing [14]. The 3D printing process is a promising solution to generate dental models out of polymers based on lab-side or intraorally acquired STL files [15]. Dental models reconstructed by 3D printing were considered clinically acceptable in terms of accuracy and reproducibility compared to classical stone casts [16]; while compared to CAD/CAM milling, 3D-printed models demonstrated even higher accuracy [17].

Three-dimensional printing is a relatively new technique in dentistry, and consequently, detailed information on the dimensional accuracy and stability of 3D printed models with regard to time and storage is not available [18]. Initial laboratory studies evaluated the accuracy of 3D-printed full-arch dental models [19,20,21,22] but did not consider the impact of aging of the models.

Therefore, this in vitro study aimed to investigate the impact of model aging on the dimensional stability of 3D-printed dental models. The hypothesis was that the period of storage time has no significant influence on the accuracy of printed models.

## 2. Experimental Section

### 2.1. Study Setup

A maxillary full-arch reference model (Model ANA-4, Frasaco GmbH, Tettnang, Germany) with abutment tooth preparation for a three-unit FDP in positions 24–26 was scanned ten times using an IOS device (TRIOS Pod, version 19.2.4, 3Shape, Copenhagen, Denmark). All IOSs were performed by an experienced single operator. The IOS system was calibrated prior to each scan, and the scan strategy followed the manufacturer’s instructions. Scan data were directly exported as STL data sets (*n* = 10).

**Figure 1:**
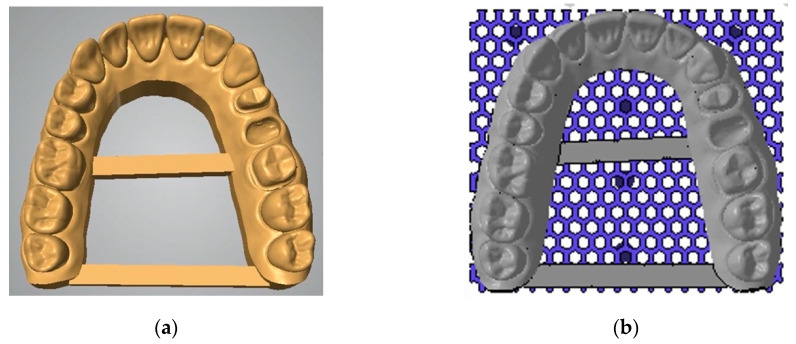
Conversion of TRIOS standard tessellation language (STL) files into 3D printable data sets with (**a**) model builder software including two stabilizing bars (**b**) virtual preparation for 3D printing using a base plate with hexagonal cell design.

Afterwards, TRIOS STL files were converted into 3D printable data sets using the built-in model builder tool of the desktop scanner (Netfabb, version 2020.2, Institut Straumann AG, Basel, Switzerland). Standardized parameters were defined for 3D-printed models with a base height of 4 mm and a thickness of 3 mm with two stabilizing bars. A base plate with hexagonal cell design with a height of 2 mm, wall thickness of 0.8 mm, and cell size of 1.5 mm was selected for all models (Figure 1).

Based on each of these IOS data sets from the TRIOS STL files, ten dental models were 3D printed (P 30, version 2019.2.11, Institut Straumann AG, Basel, Switzerland) using a light-curing 3D printing material with 385-nm wavelength technology (SHERAPrint-model plus “sand” UV, SHERA, Lemförde, Germany). This polymer is specifically formulated for the production of high-precision dental models. The printing model platform was cleaned with isopropanol and placed in the 3D printer. Afterwards, the models were removed from the platform and placed in an ultrasonic bath for cleaning. The models were then blown dry with compressed air, checked for excess material, cleaned again if required, and left to rest for 30 min before further processing. Finally, the models were exposed to a burst of light with wavelengths of 280–580 nm to cure the polymer.

The 3D-printed models were stored under constant conditions at 20 °C and 50% humidity without direct light exposure and successively digitized with laboratory desktop scanner (Series 7, version 13.1.3.33179, Institut Straumann AG, Basel, Switzerland) after storage periods of 1 day, 1 week, 2 weeks, 3 weeks, and 4 weeks.

### 2.2. Accuracy Analysis

A total of 50 STL files (ten 3D-printed dental models scanned at five timepoints) were evaluated for accuracy by means of trueness (means) and precision (standard deviations). For accuracy measurements, the original maxillary full-arch reference model was digitized using the same laboratory desktop scanner that was used to digitize the 3D-printed models after aging. Based on the manufacturer’s information, the power of the laser diode is 5 mW with a laser wavelength of 660 nm and the accuracy is specified with 7 µm.

**Figure 2:**
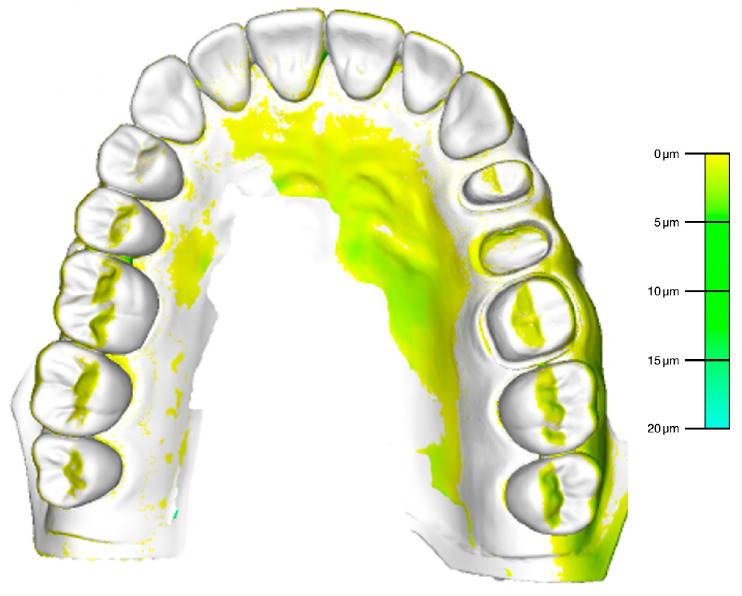
Visualization of the superimposed 3D-printed model with the reference by means of color mapping (Final Surface^®^ version 2019.0, GFaI e.V., Berlin, Germany).

The STL file of the reference model was imported into a 3D analysis software and matched pairwise with the 50 STL files of the 3D-printed models (Final Surface^®^ version 2019.0, GFaI e.V., Berlin, Germany). A best-fit algorithm of the 3D analysis software was applied for accuracy testing of the superimposed model pairings using a 2D distance analysis of the abutment teeth in areas 24 and 26 at indexed landmarks at the finishing lines. For visualization, a color mapping function of the 3D analysis software was used with a graduate scale in µm (Figure 2).

### 2.3. Statistical Analysis

Statistical analysis was carried out to evaluate the impact of aging on the accuracy of the 3D-printed dental models. Descriptive statistics were calculated for means with standard deviations (SD) including minimum and maximum values. *Wilcoxon Rank Sum Tests* were used for all comparisons. The level of significance was set at α = 0.05. Calculations were made with the open-source software “GraphPad Software” (http://www.graphpad.com).

## 3. Results

The descriptive statistics are shown in Table 1. The deviation analysis of the 3D-printed models #01–#10 compared to the reference model demonstrated overall homogenous intragroup results for distance calculations at each isolated timepoint. Taking into account the entire investigation period, the range of mean deviations was very close for all tested 3D-printed models, revealing minimum to maximum distance values of 1 to 12 µm.

**Figure 3:**
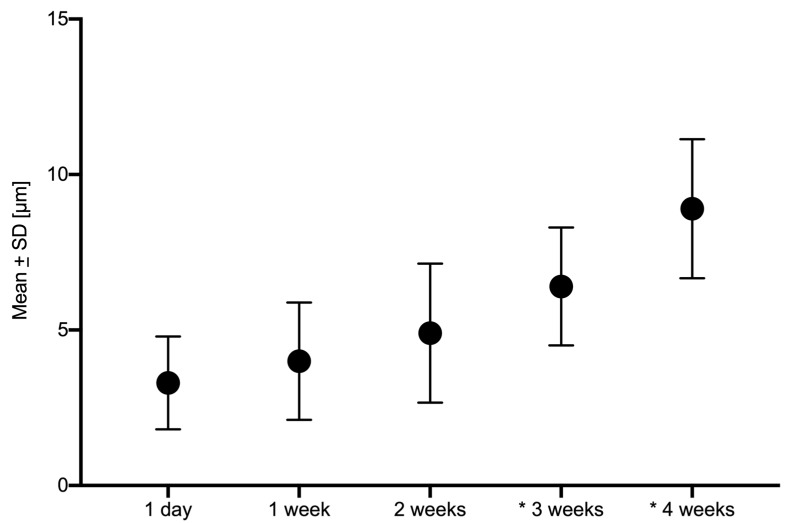
Deviation analysis of the tested 3D-printed models after defined aging represented by the five timepoints for mean values including standard deviations (SD) in micrometer (µm) with significant differences after * 3 weeks (*p* = 0.0008) and * 4 weeks (*p* < 0.0001).

Considering the factor of aging of the 3D-printed models, the most accurate result was for 1 day with a mean deviation of 3.3 ± 1.3 µm. A continuous decrease in accuracy was observed with each further aging stage of the tested 3D-printed models from 1 day up to 4 weeks (Figure 3). The time-dependent difference was statistically significant after 3 weeks (*p* = 0.0008) and 4 weeks (*p* < 0.0001), respectively, when comparing with 1 day.

## 4. Discussion

This in vitro investigation aimed to analyze the impact of aging on the dimensional stability of 3D-printed dental models. The present findings revealed a time-dependent significant change in dimensions after 3 and 4 weeks of aging. Therefore, the hypothesis that the period of storage time has no significant influence on the accuracy of 3D printed models had to be rejected.

The present study setup was carried out with an exemplary tooth-supported three-unit FDP in regions 24 to 26 for deviation analysis of the prepared abutment teeth. For fabrication of a manually veneered FDP, the overall time required-comprising the sum of clinical and technical work steps, including impression taking, try-in, potential adjustments, and seating of the reconstruction, is approximately 2–3 weeks [23]. During this period of time, the dental technician must rely on the dimensional stability of the dental cast. Based on this supposed working schedule, the present investigation considered five timepoints up to 4 weeks of aging for accuracy analysis of 3D-printed models.

The focus of the present study was to investigate the dimensional changes of the 3D-printed models related to the prepared abutment teeth representing short-span analysis rather than full-arch comparisons. The obtained results for accuracy during aging up to 4 weeks were very consistent for intragroup comparisons at each isolated timepoint. Analyzing longer spans, i.e., cross-arch reconstructions, the accuracy testing might reveal different results for intra- as well as intergroup comparisons. Even though significant differences of the 3D-printed models were observed after 3 and 4 weeks of aging, examination of the distance analysis revealed negligible changes of 1 to 12 µm for minimum to maximum values. Therefore, the question of clinical relevance remains, provided that the prosthetic reconstruction can be completed on the model within 3 or 4 weeks and that the models have been properly stored during this time (constant conditions at 20 °C and 50% humidity without direct light exposure). It must be critically emphasized that the dimensional changes of the 3D-printed models were small and comparable to investigations analyzing the accuracy of dental stone casts obtained from classical impression taking [24,25].

Ideally, dental master casts for the manufacturing of retrievable prosthetic reconstructions, such as screw-retained implant-supported FDPs or removable dental prostheses, can be reused in case of a potential emergency in the future. Tested 3D-printed models demonstrated a continuous decrease of dimensional stability. We cannot extrapolate beyond the 4-week duration of this study; therefore, it is not possible to predict with certainty whether these changes will continue or stabilize somehow over time. Thus, 3D-printed dental master models should be considered as single-use product, at least for the manufacturing of definitive prosthetic reconstructions. Nevertheless, the existing digital data sets can be stored and reused for the production of “fresh” dental models if necessary.

The bottleneck of accuracy is the process chain of STL files. The interface management guaranteeing a loss-free data transfer from the IOS device to the software of the virtual model builder and to the 3D printer is the key for success. The findings are therefore only representative for the combination of the equipment and materials used in this investigation and cannot be transferred to workflows of other manufacturers. Further investigations are necessary to analyze different setups of IOS > software > 3D printer including materials used and additional prosthetic indications. The following has to be considered: what are the impacts of the polymer and the 3D-printing technology, or is it a combination of both?

In general, the translation of laboratory findings into clinical (routine) protocols must proceed with caution. In the present study, the in vitro study setting can only be transferred with digital impressions in the upper jaw. It must be taken into account that in vivo digital impressions are different considering the localization. In contrast to the maxilla, the mandible exhibits characteristic inherent mobility during dynamic movements, in particular during mouth opening. The transfer of laboratory results from full-arch scans to an in vivo patient situation is difficult, specifically in the mandible.

While in the past, conventional workflows with classical impression techniques and plaster model production have been continuously optimized, there are still no explicit recommendations for digital workflows with IOS, further STL processing, and consecutive fabrication of 3D-printed dental models. Due to the various commercially available 3D printers with different quality levels and the diverse light-curing polymers in the material segment, the field of digitally produced dental models is very complex and subject to constant change without generally defined standards [15,26]. Kim et al. (2018) reported on significant differences for the analysis of full-arch dental models manufactured with different 3D printing techniques [27]. In addition, Nestler et al. (2020) have shown that inexpensive 3D printers were no less accurate than more expensive ones [19]. However, the authors did not clarify what is the meaning of “inexpensive” compared to “expensive”, especially when a global market is considered. Three-dimensional printing generates not only enthusiasm but also great uncertainty. Therefore, future research must focus on the definition and establishment of evidenced-based standards in the field of 3D printing techniques in dentistry. Otherwise it is not possible for both dental technicians and dentists to distinguish which workflows with which equipment and material combination can deliver reliable results [28].

## 5. Conclusions

Three-dimensionally printed dental models for the production of three-unit FDPs demonstrated very accurate results. However, a significant decrease in dimensional stability of the models was observed after 3 weeks of aging under constant conditions. Based on these findings, it can be concluded that 3D-printed dental master models should not be used for the fabrication of definitive prosthetic reconstructions more than 3 to 4 weeks after 3D printing. Nevertheless, the changes observed due to aging in this study were small and comparable to the variation seen in conventional plaster cast models that are used in routine practice today.

## Figures and Tables

**Table 1:** Deviation (in μm) of the 3D-printed models #01–#10 from the reference model after aging of 1 day; 1 week; and 2, 3, and 4 weeks (SD = standard deviation, Min = minimum, and Max = maximum).

	1 Day	1 Week	2 Weeks	3 Weeks	4 Weeks
#01	2	2	3	5	6
#02	1	2	2	3	5
#03	2	3	2	7	8
#04	2	2	3	4	9
#05	4	3	6	6	9
#06	4	6	5	7	10
#07	5	7	6	8	12
#08	5	5	7	7	9
#09	3	4	7	8	9
#10	5	6	8	9	12
Mean	3.3	4.0	4.9	6.4	8.9
SD	1.3	1.9	2.2	1.9	2.2
Min	1	2	2	3	5
Max	5	7	8	9	12
